# Exploring the acceptability of seaweed consumption among pregnant individuals

**DOI:** 10.1177/02601060251401628

**Published:** 2025-12-08

**Authors:** Anne-Laurence Landry, Marianne Rochette, Emilie Bernier, Inès Auclair-Mangliar, Anne-Sophie Plante, Véronique Provencher, Lamia L’Hocine, Lucie Beaulieu, Anne-Sophie Morisset

**Affiliations:** 1 School of Nutrition, 4440 Université Laval, Quebec City, Canada; 2 Endocrinology and Nephrology Unit, 36896 CHU of Québec-Laval University, Quebec City, Canada; 3 Centre Nutrition, Santé et Société (NUTRISS), INAF, Université Laval, Quebec City, Canada; 4 Agriculture and Agri-Food Canada, 6337 Saint-Hyacinthe Research and Development Centre, Saint-Hyacinthe, Canada; 5 Institute on Nutrition and Functional Foods, Université Laval, Quebec City, Canada; 6 Department of Food Science, Université Laval, Quebec City, Canada; 7 Québec-Océan, Université Laval, Quebec City, Canada

**Keywords:** Seaweeds, algae, perceptions, food, pregnancy

## Abstract

**Background:**

Seaweed is a nutrient-dense, sustainable, and underutilized food source. Despite its growing popularity, little is known about its consumption and related perceptions during pregnancy.

**Aim:**

To (1) explore perceptions of health, environmental concerns, and seaweed consumption among pregnant individuals; (2) characterize their seaweed consumption patterns; and (3) identify factors shaping their attitudes and choices regarding seaweed.

**Methods:**

Data were collected via an online survey developed for this study, incorporating items from the Health Consciousness Scale and questions assessing perceptions, consumption patterns, and attitudes related to seaweed among 120 pregnant participants across all trimesters. Descriptive and qualitative analyses were conducted.

**Results:**

In terms of health perceptions, most respondents (91.7%) reported being actively engaged in their health, and a majority (81.7%) agreed that the healthfulness of foods greatly influences their dietary choices. Regarding seaweed consumption, 70.8% of participants had consumed seaweed at least once during pregnancy. In addition, 60.0% of respondents expressed a positive attitude toward seaweed.

**Conclusions:**

Incorporating seaweed into dietary recommendations during pregnancy may offer both nutritional and environmental benefits. Future research should prioritize rigorous safety assessments to establish evidence-based guidance for seaweed consumption among pregnant individuals.

## Introduction

According to the United Nations, the world population is expected to reach 9.7 billion in 2050 (United Nations, 2019), creating a pressing need to increase food production by approximately 50%, compared to 2012 levels (FAO, 2017). Achieving this will require the widespread adoption of sustainable agricultural practices to enhance productivity without compromising environmental resources (FAO, 2017). In this context, seaweed represents a promising and underutilized food source that could contribute to sustainable nutrition.

Seaweed, or macroalgae, offers several advantages over terrestrial crops. They grow in marine environments, do not require fresh water or arable land, and can achieve higher yield per area than many land-based plants (Bleakley and Hayes, 2017; Van Krimpen et al., 2013). Their consumption is popular in several Asian cultures (Murai et al., 2021) and is gaining interest in Western countries—including Canada—namely due to its high nutritional value, balanced amino acid profile, and sustainable production potential (Afonso et al., 2019; Salehi et al., 2019; Tremblay and Beaulieu, 2021). Canada, particularly the province of Quebec, offers favorable conditions for the development of local seaweed industries, with extensive cold-water coastlines, low levels of marine pollution, and a growing interest in sustainable food systems (Dupré-Gilbert and Berger, 2018).

Nutritionally, seaweeds are rich in protein, fiber, vitamins, and minerals, although content varies by species and geographic origin (Becker, 2007; Fleurence et al., 2012). Red and green seaweeds can contain approximately 6 g of protein per 100 g of fresh weight, which is higher than common vegetables, such as spinach or broccoli, while brown seaweeds generally have lower protein content (Bleakley and Hayes, 2017; Health Canada, 2015; Holdt and Kraan, 2011). Seaweeds are also valuable sources of micronutrients, including iodine, iron, magnesium, calcium, potassium, and sodium, as well as folic acid and vitamins A, C, and K (Marfaing, 2017; Mouritsen et al., 2012; Salehi et al., 2019).

During pregnancy, nutritional requirements increase, particularly for micronutrients such as folic acid, iron, and calcium, which play key roles in maternal and fetal health (WHO, 2016). Due to their nutrient density, seaweeds have the potential to support increased nutritional demands during pregnancy. Nevertheless, certain species may contain high levels of iodine and sodium, which, if consumed excessively, can contribute to health risks such as hypo- or hyperthyroidism and hypertension (Circuncisão et al., 2018; Salehi et al., 2019). For example, sodium content can range from 414 to 14,350 mg per 100 g of dry weight, depending on species and origin (Pereira, 2018). Furthermore, brown seaweed can contain up to 624,500 µg iodine per 100 g of dry weight (Cherry et al., 2019; Zimmermann and Delange, 2004), far exceeding the pregnancy tolerable upper intake level of 1100 µg, even if consumed in small quantities (Otten et al., 2006). These variations underscore the importance of species-specific and context-specific consumption guidance, particularly for vulnerable populations such as pregnant individuals.

Recommendations for seaweed consumption during pregnancy vary worldwide. A joint report by the Food and Agriculture Organization and the World Health Organization recently highlighted the growing importance of seaweed for sustainable food systems, while also noting potential food safety hazards and the lack of formal guidance, especially for high-risk groups (FAO and WHO, 2022). In countries such as France and Germany, seaweed consumption is discouraged for pregnant and breastfeeding individuals due to potential risks associated with high iodine content and other contaminants (ANSES, 2018; Koletzko et al., 2013). In other regions, moderate consumption is permitted, often with restrictions based on seaweed type. For instance, in Australia and New Zealand, pregnant and breastfeeding individuals are advised to consume no more than one serving per week of brown seaweed not only to prevent excessive iodine intakes, but also to limit exposure to potential contaminants, such as arsenic, mercury, and lead (FSANZ, 2011). In Japan, a country with traditionally high seaweed intake, no specific recommendations prohibit consumption during pregnancy, although some studies suggest that frequent consumers may be exposed to contaminants (Mise et al., 2019; Xiao et al., 2024). In Canada, no formal guidance exists regarding seaweed consumption during pregnancy.

Given this context, exploring pregnant individuals’ perceptions, consumption patterns, and potential safety concerns regarding seaweed consumption is essential to inform evidence-based recommendations. Therefore, this study aims to (1) explore perceptions of health, environmental concerns, and seaweed consumption among pregnant individuals; (2) characterize their seaweed consumption patterns; and (3) identify factors shaping their attitudes and choices regarding seaweed.

## Materials and methods

### Participant eligibility criteria and recruitment

This cross-sectional study targeted pregnant individuals from the Quebec City area. Eligible participants were at least 18 years old and pregnant, regardless of gestational age, and able to access and complete an online survey in French. Recruitment and data collection occurred concurrently between September 2022 and May 2023. Participants were recruited through various channels, including social media, email, posters, and a collaboration with registered dietitians at the *Centre Hospitalier Universitaire (CHU) de Québec-Université Laval*. Upon accessing the study webpage via QR code or a web link, potential participants were first presented with an information and consent form describing the study's objectives, procedures, voluntary nature, anonymity, and ethics approval by the CHU de Québec-Université Laval Research Ethics Board. The preamble to the consent form explicitly stated the eligibility criteria. Individuals who consented were then automatically directed to the online survey.

This project was approved by the CHU de Québec-Université Laval Research Center's Ethics Committee (Reference number: 2023-6157). All participants provided informed consent before answering the survey.

### Survey design and development

Due to the lack of validated instruments available to specifically assess factors influencing seaweed consumption during pregnancy, we developed an online survey based on established measures of attitudes toward food, health, and environment, as well as on evidence-based literature on dietary behaviors in pregnant populations. The original French version of the questionnaire, as well as an English translation, are available in the Supplementary materials.

The development of the survey followed a systematic process. First, a comprehensive literature review was conducted to identify key constructs and relevant items regarding dietary attitudes, health consciousness, food neophobia, and environmental awareness in pregnant populations. Items from validated scales, including the Health Consciousness Scale (Gould, 1990), the Health and Taste Scales (Roininen et al., 1999), and the Food Neophobia Scale (Pliner and Hobden, 1992; Ritchey et al., 2003), were selected and carefully adapted to ensure contextual relevance for pregnancy. Questions were logically ordered, and response options were standardized to reduce respondent burden and minimize potential measurement bias. The survey was designed to be concise, engaging, and user-friendly, with an estimated completion time of 10 minutes. The final 23-item survey was organized into three main sections: (1) participant characteristics; (2) general attitudes toward food and health; and (3) seaweed consumption and perceptions.

The first section included six questions on participants’ characteristics, including age, gestational age, annual household income, education status, and their usual and cultural dietary practices.

The second section included three questions and assessed participants’ general attitude toward food and health during pregnancy, using 5-point Likert scales ranging from “Strongly disagree” to “Strongly agree.” The first question consisted of four sub-items measuring health consciousness and attitudes toward the healthfulness of foods, adapted from the Health Consciousness Scale (Gould, 1990) and the Health and Taste Scales (Roininen et al., 1999). The second question consisted of three sub-items measuring the participant's attitude toward new or unfamiliar foods, adapted from the Food Neophobia Scale (Pliner and Hobden, 1992; Ritchey et al., 2003). The final question evaluated awareness of the environmental impact of individual food choices.

The third section focused specifically on seaweed consumption and perceptions and included a brief definition of seaweeds, providing participants with basic contextual information. This section comprised 14 specific questions. Three questions assessed participants’ attitudes toward seaweed using a 3-point ordinal scale (“Negative,” “Neutral,” or “Positive”) to capture their overall perception and evaluation of the food and its potential health effects. Two questions examined perceived risks associated with seaweed consumption for the participant and their baby, using a 3-point ordinal scale (“Yes,” “Maybe,” or “No”) to capture the perceived likelihood of adverse effects. One binary question assessed whether participants had consumed seaweed during pregnancy. This section was followed by six questions directed specifically to participants who reported having consumed seaweed. These questions explored the forms consumed, frequency and portion sizes, motivations for consumption, and intentions to continue consuming seaweed during pregnancy. Two additional questions addressed reasons for not consuming seaweed and willingness to try it, particularly among those who had never eaten it. Finally, an open-ended question provided participants the opportunity to share any additional concerns or perspectives regarding seaweed consumption during pregnancy.

### Survey administration and data collection

The survey was hosted on LimeSurvey (LimeSurvey GmbH, Hamburg, Germany), enabling participants to complete the questionnaire online and anonymously. The system recorded start and end times for each response and prevented multiple submissions from the same participant. Data were exported in anonymized format for analysis, ensuring confidentiality and secure handling. This allowed us to track completion rates, monitor survey progress, and ensure data integrity.

Participant inclusion is illustrated in a flowchart in the Supplemental materials (Figure S1). Of the 168 individuals who accessed the study webpage, 120 provided informed consent, and completed the survey. No participants withdrew after providing consent.

### Sample size

Given the exploratory nature of this study, no formal sample size calculation was performed. The target sample was determined based on feasibility, considering recruitment reach and anticipated response rates, and was deemed sufficient for descriptive summaries and qualitative analyses.

### Statistical analyses

Quantitative data analysis was performed using the LimeSurvey platform (LimeSurvey GmbH, Hamburg, Germany; version 3.25.20.210330). Given the exploratory nature of the study, analyses were limited to descriptive statistics, including means and standard deviations for continuous variables and frequencies and percentages for categorical variables.

Qualitative analyses were conducted separately on responses to open-ended questions to explore participants’ perspectives in greater depth. Two researchers independently applied a thematic sub-grouping approach, categorizing responses based on emerging ideas and recurring patterns. Discrepancies were discussed and resolved by consensus to enhance analytical rigor and reliability.

## Results

### Characteristics of participants

A total of 120 pregnant individuals completed the online survey in approximately 4 ± 2 minutes. Respondents were aged 30.8 ± 3.9 (range: 18–43) years old and were at 24.2 ± 9.4 (range: 6–38) weeks of pregnancy (Table 1). Most of the sample had an annual household income of CAD$100,000 or more and had a university degree. Regarding dietary patterns, 87.5% described themselves as omnivores, 11.7% as vegetarians, and 0.8% as vegan.

**Table 1.** Participant's characteristics (*n* = 120).

Variables	Mean ± SD or *n* (%)
Age (years)	30.8 ± 3.9
Gestational weeks	24.2 ± 9.4
Annual household income, CAD$^a^	
0–49,999	12 (10.0)
50,000–99,999	40 (33.3)
100,000 or more	63 (52.5)
Prefer not to answer	5 (4.2)
Education level	
High school diploma or less	7 (5.8)
Diploma of vocational studies	4 (3.3)
College diploma or sub-bachelor's degree certificate	31 (25.8)
Bachelor's degree or beyond	78 (65.0)
Food pattern	
Omnivore	105 (87.5)
Vegetarian	14 (11.7)
Vegan	1 (0.8)
Seaweeds are a part of dietary traditions or culture	
Yes	43 (35.8)
No	77 (64.2)

a In Canada, in 2024, federal minimum wage is $17.30/h.

### Attitudes toward health and food

A majority of respondents reported being engaged in their health (91.7%) and aware of their health status during pregnancy (97.5%) (Table 2). Most participants (81.7%) agreed or strongly agreed that the healthfulness of foods substantially influenced their dietary choices, and 62.5% indicated paying close attention to the nutritional content of the foods consumed during pregnancy. Notably, 67.5% of respondents reported being aware of the environmental impact of their dietary choices.

**Table 2.** Attitudes toward health and food during pregnancy (*n* = 120).

	Strongly disagree	Disagree	Neutral	Agree	Strongly agree
I am very involved in my health	0 (0.0)	1 (0.8)	9 (7.5)	57 (47.5)	53 (44.2)
I am very aware of my health	0 (0.0)	0 (0.0)	3 (2.5)	59 (49.2)	58 (48.3)
The health aspect of foods greatly influences my food choices	1 (0.8)	3 (2.5)	18 (15.0)	66 (55.0)	32 (26.7)
I pay very close attention to the nutritional values of the foods I eat	4 (3.3)	10 (8.3)	31 (25.8)	61 (50.8)	14 (11.7)
I am aware of the impact of my food choices on the environment	3 (2.5)	6 (5.0)	30 (25.0)	63 (52.5)	18 (15.0)
I am willing to try new foods	3 (2.5)	27 (22.5)	18 (15.0)	54 (45.0)	18 (15.0)
I am afraid of eating foods that I have never tried before	19 (15.8)	45 (37.5)	14 (14.2)	33 (27.5)	6 (5.0)

### Seaweed consumption patterns

Most respondents (70.8%) had consumed seaweed during their pregnancy (Table 3). The most common form was sushi (85.9%), followed by dried chips or nori sheets (21.2%), salads (18.8%), and soups or broths (15.3%). The frequency of consumption varied, with 49.4% consuming seaweed a few times during pregnancy, 30.6% consuming it 3 times or less per month, and 9.4% consuming it once a week or more. The main reasons for consumption were taste (69.4%) and dietary variety (17.6%). While most participants (55.3%) planned to consume seaweed again during their pregnancy, 35.3% were undecided, and 8.2% did not intend to consume it again.

**Table 3.** Seaweed consumption patterns (*n* = 120).

	*n* (%)
Seaweed consumption (yes)	85 (70.8)
Seaweed forms consumed	
Sushis	73 (85.9)
Dried	18 (21.2)
Salads	16 (18.8)
Soup	13 (15.3)
Other forms	18 (21.2)
Frequency of seaweed consumption^a^	
Once a week or more	8 (9.4)
3 times or less per month	26 (30.6)
Few times during pregnancy	42 (49.4)
Once during pregnancy	9 (10.6)
Amount consumed on a single occasion^a^	
5–10 mL	11 (12.9)
15–30 mL	26 (30.6)
60 mL	25 (29.4)
125 mL	11 (12.9)
190 mL	1 (1.2)
Other	11 (12.9)
Reasons of eating seaweed^a^	
For taste	59 (69.4)
To make it different, for variety	15 (17.6)
For health-related benefits	3 (3.5)
By tradition	3 (3.5)
Because it comes with sushis	3 (3.5)
All of these answers	2 (2.4)
Planning to eat seaweed again during pregnancy^a^	
Yes	47 (55.3)
Maybe	30 (35.3)
No	7 (8.2)
No answer	1 (1.2)
Willingness to try seaweed during pregnancy^b^	
Yes	10 (28.6)
Maybe	14 (40.0)
No	11 (31.4)

a *n* = 85.

b *n* = 35.

### Perceptions regarding seaweeds and factors influencing dietary choices

More than half of respondents (60.0%) reported a positive attitude toward seaweed, with 43.3% believing that eating seaweed had beneficial effects on their health (Table 4). Very few participants (0.8%) associated seaweed consumption with personal health risks. Regarding potential fetal risks, 65.8% perceived no risk, 30.0% were undecided, and 3.3% believed there were risks.

**Table 4.** Perceptions and attitudes regarding seaweed consumption (*n* = 120).

	Negative	Neutral	Positive
What is your attitude toward seaweed?	7 (5.8)	41 (34.2)	72 (60.0)
Would you say that eating seaweed has negative or positive effects for your health?	1 (0.8)	67 (55.8)	52 (43.3)
Would you say that eating seaweed has negative or positive effects on the health of your baby?	4 (3.3)	86 (71.7)	30 (25.0)

	No	Maybe	Yes
Do you believe that eating seaweed is associated with risks for your health?	93 (77.5)	26 (21.7)	1 (0.8)
Do you believe that eating seaweed is associated with health risks for your baby?	79 (65.8)	36 (30.0)	4 (3.3)

Before pregnancy, 75.0% of participants agreed or strongly agreed that they often tried new foods (data not shown). In contrast, during pregnancy, openness to trying new foods was slightly lower, with 60.0% of participants agreeing or strongly agreeing that they were willing to try new foods (Table 2). Conversely, regarding fear of trying unfamiliar foods during pregnancy, 53.3% of participants disagreed or strongly disagreed with the statement, while 32.5% agreed or strongly agreed, and 14.2% were neutral (Table 2).

Regarding qualitative data on factors influencing dietary choices, 25 of the 120 participants (20.8%) expressed concerns and opinions related to seaweed consumption during pregnancy.

Safety and preparation were central themes, encompassing potential contamination, high iodine content, and risks of foodborne illness. One participant explained “I would tend to cook the seaweed prepared at home thoroughly to be sure that it is good to eat when pregnant. Not necessarily for the algae as such, but for the bacteria/viruses that may be found on it.” These safety considerations were often linked to habitual consumption practices, as seaweed is frequently eaten raw or dried. As one participant noted, “Seaweed is often eaten raw or dried (without cooking). We are recommended to eat cooked foods.”

A lack of knowledge also influenced participants’ decisions. Some had never verified whether seaweed is recommended during pregnancy, and others indicated that additional information would have encouraged them to include it in their diet. One participant reflected: “Perhaps with more information and more studies, I would have integrated this food.” Several participants reported that they would seek guidance if they planned to increase consumption or try seaweed for the first time during pregnancy.

Nutritional considerations further shaped participants’ perceptions. Some highlighted the health benefits and micronutrient content of seaweed, with one participant stating, “I was already consuming it for its benefits and iron content, and I continued during my first pregnancy as well as the current one.” Others were attentive to potential risks related to nutrient content, such as sodium, with one noting, “One concern could be the amount of salt in relation to blood pressure.”

Other factors, including concerns about the origin and quality of seaweed, but also pregnancy-related aversions or sickness influenced participants’ choices. One participant explained, “I had an immediate aversion [to seaweed] around 6–7 weeks of pregnancy. I haven’t tried it again since, as I’m still dealing with first-trimester nausea. It's not a health concern, but rather an aversion.”

## Discussion

To our knowledge, this is the first study to examine perceptions and consumption of seaweed among pregnant individuals. Our findings indicate that most participants considered health as an important factor influencing their dietary choices. Most participants had consumed seaweed and intended to consume it again during pregnancy. While attitudes toward seaweed were generally positive, some participants expressed specific concerns regarding its safety during pregnancy.

### Attitudes toward health and food during pregnancy

Our findings indicate that a large proportion of participants expressed a strong engagement with their health during pregnancy and acknowledged that the healthfulness and nutritional content of foods significantly guided their decisions regarding food consumption. This is consistent with the findings of a study conducted by Ferraro et al., in Ottawa, Canada and further supported by more recent studies demonstrating that pregnant individuals express a strong desire and motivation to adopt healthier eating habits as soon as pregnancy is confirmed (Ferraro et al., 2011; Gagnon et al., 2023; Poon et al., 2018). Additionally, qualitative research identified the desire for good health as a key factor influencing eating behavior throughout pregnancy (Rockliffe et al., 2022). These results suggest that health considerations both for themselves and their fetus play a crucial role in the dietary choices of pregnant individuals.

Our results also revealed that participants were generally aware of the environmental impact of their food choices, reflecting the growing relevance of sustainable and conscious consumption. Seaweed, in particular, offers important environmental benefits (Campbell et al., 2019), as it requires few resources compared to intensive agriculture, while also providing a nutrient-rich profile, including a high protein content, dietary fibers, micronutrients, and bioactive compounds (Holdt and Kraan, 2011; Shannon and Abu-Ghannam, 2019). Thus, participants’ choices regarding the consumption of seaweed appear to be influenced by both its health and environmental attributes.

### Seaweed consumption patterns

Although seaweed is not traditionally part of the dietary culture of most participants, its consumption—primarily in the form of sushi—was nevertheless relatively common. Several factors may explain this finding. First, familiarity is known to shape food acceptance (Hoek et al., 2011) and has been specifically shown to influence seaweed consumption (Onwezen et al., 2021). Second, exposure has likely been reinforced by the growing popularity and accessibility of Asian cuisine in Canada, reflecting a broader cultural globalization (Ferdouse et al., 2018; Gao, 2022; Statistics Canada, 2022). Third, local initiatives and culinary workshops available in the province of Quebec may also have contributed to increased awareness and consumption of seaweed products (Lafeuille et al., 2024; Maillard et al., 2023). At the same time, dietary guidelines during pregnancy often recommend avoiding raw foods such as fish, which could limit sushi consumption (Health Canada, 2021; Super and Wagemakers, 2021). However, this influence may have been less pronounced in our sample, since participants had relatively high levels of education and household income—factors generally associated with heightened attention to food quality and a preference for foods perceived as both safe and healthy (Darmon and Drewnowski, 2015; Lallukka et al., 2007). In summary, while seaweed is not a cultural staple of pregnant individuals from Quebec, its consumption was relatively frequent in our study population. This pattern appears to result from a combination of increased exposure through globalization and local initiatives, balanced against pregnancy-specific dietary recommendations, with socioeconomic characteristics of participants likely shaping their overall practices.

### Perceptions regarding seaweeds and factors influencing dietary choices

This study also revealed a generally positive attitude toward seaweed consumption among participants, with 60.0% expressing a favorable outlook and 43.3% recognizing potential health benefits. These perceptions are consistent with the growing evidence supporting seaweed as a nutritious and sustainable food (Afonso et al., 2019; Salehi et al., 2019).

Concerns about potential risks were less prevalent, as only a minority believed that seaweed consumption could pose health issues for themselves or their future children. Nonetheless, seaweed may accumulate iodine and heavy metals depending on its growing environment. Iodine content is highly variable across species, ranging from relatively modest levels in red and green algae to extremely high concentrations in some brown seaweed, sometimes well above the tolerable upper intake of 1100 µg/day during pregnancy. Excessive iodine intake during pregnancy or breastfeeding has been associated with a higher risk of elevated thyroid-stimulating hormone levels or hypothyroidism in infants, or other adverse outcomes (Murai et al., 2021). Processing methods such as blanching can reduce iodine content, enabling greater consumption without exceeding tolerable upper intake levels, though they may also decrease beneficial compounds such as minerals, carotenoids, and polyphenols (Lafeuille et al., 2023). These findings highlight the need for evidence-based guidelines to establish safe consumption limits during pregnancy.

Our results also showed a reduction in the willingness to try new foods during pregnancy, declining from 75.0% before pregnancy to 60.0% during pregnancy. This shift likely reflects heightened health consciousness, reinforced by regular counseling from healthcare professionals (Lott et al., 2019), as well as internal factors such as food cravings, aversions, and gastrointestinal symptoms (Paupério et al., 2014). These factors, combined with limited information on seaweed noted by some participants, may contribute to the reluctance toward unfamiliar foods. Similarly, previous research indicated that young Australian consumers expressed a desire for greater awareness and knowledge about seaweed (Young et al., 2022). Overall, these findings emphasize that while seaweed is generally perceived positively, information gaps and pregnancy-related dietary caution can limit their consumption. Addressing these multifaceted barriers through tailored nutritional guidance could support informed dietary choices in this population.

### Strengths and limitations

This study has several strengths and limitations. The use of an online questionnaire may have encouraged participants to respond more openly, thereby reducing social desirability bias and allowing us to capture perceptions and behaviors in a relatively understudied field. However, some limitations must be acknowledged. The sample was small, relatively homogeneous, and characterized by high socioeconomic and education levels, which limits the generalizability to more diverse populations. In addition, the survey relied partly on items adapted from English-language tools, which may have introduced measurement bias. Despite these constraints, the study provides meaningful and original insights into seaweed consumption and perceptions during pregnancy, and highlights the need for future research including more diverse populations and validated instruments.

## Conclusion

Our findings suggest that a large proportion of respondents expressed a positive attitude toward seaweed, as most participants reported being health-conscious and indicated prior consumption of seaweed during pregnancy. Taken together, these observations highlight that incorporating seaweed into nutritional recommendations during pregnancy follow-ups could be a promising strategy, given its notable nutritional value, low environmental footprint, and potential to diversify diet. However, the safety of seaweed consumption during pregnancy requires careful verification, particularly regarding heavy metal concentrations and microbial contamination. It is therefore essential that healthcare professionals, including dietitians and physicians, provide evidence-based information on both the potential risks and benefits of seaweed consumption. Future research should prioritize rigorous safety assessments to established evidence-based recommendations for pregnant individuals. Finally, harmonizing international regulations on seaweed contaminants, particularly for imported products, would help secure consistent safety standards and minimize exposure risks for vulnerable populations, including pregnant individuals.

## Supplemental Material

sj-docx-1-nah-10.1177_02601060251401628 - Supplemental material for Exploring the acceptability of seaweed consumption among pregnant individuals Supplemental material, sj-docx-1-nah-10.1177_02601060251401628 for Exploring the acceptability of seaweed consumption among pregnant individuals by Anne-Laurence Landry, Marianne Rochette, Emilie Bernier, Inès Auclair-Mangliar, Anne-Sophie Plante, Véronique Provencher, Lamia L’Hocine, Lucie Beaulieu and Anne-Sophie Morisset in Nutrition and Health

## Data Availability

The data presented in this study are available on request from the corresponding author.

## References

[bibr1-02601060251401628] Afonso NC Catarino MD Silva AMS , et al. (2019) Brown macroalgae as valuable food ingredients. Antioxidants 8(9): 65. 10.3390/antiox8090365 PMC6769643 31480675

[bibr2-02601060251401628] ANSES (2018) Opinion of the French Agency for Food, Environmental and Occupation Health and Safety (ANSES) on the risk of excess iodine intake from the consumption of seaweed in foodstuffs. Maisons-Alfort, France: Agence nationale de sécurité sanitaire de l’alimentation, de l’environnement et du travail.

[bibr3-02601060251401628] Becker EW (2007) Micro-algae as a source of protein. Biotechnology Advances 25(2): 207–210. 10.1016/j.biotechadv.2006.11.002 17196357

[bibr4-02601060251401628] Bleakley S Hayes M (2017) Algal proteins: Extraction, application, and challenges concerning production. Foods (Basel, Switzerland) 6(5): 33. 10.3390/foods6050033 PMC5447909 28445408

[bibr5-02601060251401628] Campbell I Macleod A Sahlmann C , et al. (2019) The environmental risks associated with the development of seaweed farming in Europe—Prioritizing key knowledge gaps. Frontiers in Marine Science 6: Article 07.

[bibr6-02601060251401628] Cherry P O'Hara C Magee PJ , et al. (2019) Risks and benefits of consuming edible seaweeds. Nutrition Reviews 77(5): 307–329. 10.1093/nutrit/nuy066 PMC6551690 30840077

[bibr7-02601060251401628] Circuncisão AR Catarino MD Cardoso SM , et al. (2018) Minerals from macroalgae origin: Health benefits and risks for consumers. Marine Drugs 16(11): 400. 10.3390/md16110400 PMC6266857 30360515

[bibr8-02601060251401628] Darmon N Drewnowski A (2015) Contribution of food prices and diet cost to socioeconomic disparities in diet quality and health: A systematic review and analysis. Nutrition Reviews 73(10): 643–660. 10.1093/nutrit/nuv027 PMC4586446 26307238

[bibr9-02601060251401628] Dupré-Gilbert E Berger K (2018) *Stratégie Algues*. https://merinov.ca/wp-content/uploads/2021/10/Merinov-Strategie-Algues.pdf.

[bibr10-02601060251401628] FAO (2017) The Future of Food and Agriculture—Trends and Challenges. Rome: Food and Agriculture Organization of the United Nations.

[bibr11-02601060251401628] FAO and WHO (2022) Report of the Expert Meeting on Food Safety for Seaweed—Current Status and Future Perspectives. Rome, 28–29 Octobre 2021. Food Safety and Quality Series. Rome: Food and Agriculture and Organization of the United Nations & World Health Organization.

[bibr12-02601060251401628] Ferdouse F Løvstad Holdt S Smith R , et al. (2018) The global status of seaweed production, trade and utilization. FAO Globefish Research Programme 124: 120pp.

[bibr13-02601060251401628] Ferraro Z Rutherford J Keely EJ , et al. (2011) An assessment of patient information channels and knowledge of physical activity and nutrition during pregnancy. Obstetric Medicine 4(2): 59–65. 10.1258/om.2011.110006 PMC4989737 27582855

[bibr14-02601060251401628] Fleurence J Morançais M Dumay J , et al. (2012) What are the prospects for using seaweed in human nutrition and for marine animals raised through aquaculture? Trends in Food Science & Technology 27(1): 57–61.

[bibr15-02601060251401628] FSANZ (2011) *Consumer—Food safety and recalls—Brown seaweed—Advice for pregnant women, breastfeeding women and children* . In: Food Standards Australia New Zealand. Canberra, Australia: Food Standards Australia New Zealand. www.foodstandards.gov.au/consumer/safety/brownseaweed/pages/default.aspx .

[bibr16-02601060251401628] Gagnon M Plante AS Turcotte M , et al. (2023) Determinants of weight gain and body changes during pregnancy: A qualitative study of French-Canadian pregnant people. Journal of Gynecology Obstetrics and Human Reproduction 52(2): 102519. 10.1016/j.jogoh.2022.102519 36529367

[bibr17-02601060251401628] Gao R (2022) Cuisine chinoise au Canada. https://www.thecanadianencyclopedia.ca/fr/article/cuisine-chinoise-au-canada.

[bibr18-02601060251401628] Gould SJ (1990) Health consciousness and health behavior: The application of a new health consciousness scale. American Journal of Preventive Medicine 6(4): 228–237. 2223170

[bibr19-02601060251401628] Health Canada (2015) Canadian Nutrient File (CNF). Ontaria, Canada: Government of Canada. Available at: https://food-nutrition.canada.ca/cnf-fce/.

[bibr20-02601060251401628] Health Canada (2021) *Food safety for pregnant women*. Available at: https://www.canada.ca/en/health-canada/services/food-safety-vulnerable-populations/food-safety-pregnant-women.html.

[bibr21-02601060251401628] Hoek AC Luning PA Weijzen P , et al. (2011) Replacement of meat by meat substitutes. A survey on person- and product-related factors in consumer acceptance. Appetite 56(3): 662–673. 10.1016/j.appet.2011.02.001 21315123

[bibr22-02601060251401628] Holdt SL Kraan S (2011) Bioactive compounds in seaweed: Functional food applications and legislation. Journal of Applied Phycology 23(3): 543–597.

[bibr23-02601060251401628] Koletzko B Bauer CP Bung P , et al. (2013) German national consensus recommendations on nutrition and lifestyle in pregnancy by the “healthy start—young family network”. Annals of Nutrition & Metabolism 63(4): 311–322. 10.1159/000358398 24514069

[bibr24-02601060251401628] Lafeuille B Tamigneaux E Berger K , et al. (2023) Variation of the nutritional composition and bioactive potential in edible macroalga Saccharina latissima cultivated from Atlantic Canada subjected to different growth and processing conditions. Foods (Basel, Switzerland) 12(8): 1736. 10.3390/foods12081736. PMC10137355 37107531

[bibr25-02601060251401628] Lafeuille B Turcotte M, Tamigneaux É , et al. (2024) Increasing intention to eat seaweeds through culinary workshops with a chef: A randomized controlled study on Quebec consumers. Food Quality and Preference 113. 10.1016/j.foodqual.2023.105074.

[bibr26-02601060251401628] Lallukka T Laaksonen M Rahkonen O , et al. (2007) Multiple socio-economic circumstances and healthy food habits. European Journal of Clinical Nutrition 61(6): 701–710. 10.1038/sj.ejcn.1602583 17180154

[bibr27-02601060251401628] Lott ML Power ML Reed EG , et al. (2019) Patient attitudes toward gestational weight gain and exercise during pregnancy. Journal of Pregnancy 2019: 4176303. 10.1155/2019/4176303 PMC6766150 31637057

[bibr28-02601060251401628] Maillard É Beaulieu L Corcuff R , et al. (2023) *Les macroalgues du Québec : un atout pour la création de nouveaux aliments*. Centre de référence en agriculture et agroalimentaire du Québec. Université Laval.

[bibr29-02601060251401628] Marfaing H (2017) Qualités nutritionnelles des algues, leur présent et futur sur la scène alimentaire. Cahiers de Nutrition et de Diététique 52(5): 257–268.

[bibr30-02601060251401628] Mise N Ohtsu M Ikegami A , et al. (2019) Hijiki seaweed consumption elevates levels of inorganic arsenic intake in Japanese children and pregnant women. Food Additives & Contaminants. Part A, Chemistry, Analysis, Control, Exposure & Risk Assessment 36(1): 84–95. 10.1080/19440049.2018.1562228 30632947

[bibr31-02601060251401628] Mouritsen GO Williams L Bjerregaard R , et al. (2012) Seaweeds for umami flavour in the new Nordic cuisine. Flavour 1(1): 4.

[bibr32-02601060251401628] Murai U Yamagishi K Kishida R , et al. (2021) Impact of seaweed intake on health. European Journal of Clinical Nutrition 75(6): 877–889. 10.1038/s41430-020-00739-8 32908248

[bibr33-02601060251401628] Onwezen MC Bouwman EP Reinders MJ , et al. (2021) A systematic review on consumer acceptance of alternative proteins: Pulses, algae, insects, plant-based meat alternatives, and cultured meat. Appetite 159. 10.1016/j.appet.2020.105058. 33276014

[bibr34-02601060251401628] Otten J Hellwig K Meyers L (2006) Dietary Reference Intakes: The Essential Guide to Nutrient Requirements. Washington DC: Institute of Medicine, National Academies Press.

[bibr35-02601060251401628] Paupério A Severo M Lopes C , et al. (2014) Could the Food Neophobia Scale be adapted to pregnant women? A confirmatory factor analysis in a Portuguese sample. Appetite 75: 110–116. 10.1016/j.appet.2013.12.023 24406850

[bibr36-02601060251401628] Pereira L (2018) Therapeutic and Nutritional Uses of Algae. 1st ed. Boca Raton, FL: CRC Press.

[bibr37-02601060251401628] Pliner P Hobden K (1992) Development of a scale to measure the trait of food neophobia in humans. Appetite 19(2): 105–120. 10.1016/0195-6663(92)90014-w 1489209

[bibr38-02601060251401628] Poon LC McIntyre HD Hyett JA , et al. (2018) The first-trimester of pregnancy—A window of opportunity for prediction and prevention of pregnancy complications and future life. Diabetes Research and Clinical Practice 145: 20–30. 10.1016/j.diabres.2018.05.002 29852233

[bibr39-02601060251401628] Ritchey PN Frank RA Hursti U-K , et al. (2003) Validation and cross-national comparison of the Food Neophobia Scale (FNS) using confirmatory factor analysis. Appetite 40(2): 163–173. 10.1016/s0195-6663(02)00134-4 12781166

[bibr40-02601060251401628] Rockliffe L Smith DM Heazell AEP , et al. (2022) A qualitative exploration of influences on eating behaviour throughout pregnancy. BMC Pregnancy and Childbirth 22(1): 39. 10.1186/s12884-022-05135-7 PMC9754306 36522706

[bibr41-02601060251401628] Roininen K LäHteenmäKi L Tuorila H (1999) Quantification of consumer attitudes to health and hedonic characteristics of foods. Appetite 33(1): 71–88. 10.1006/appe.1999.0232 10447981

[bibr42-02601060251401628] Salehi B Sharifi-Rad J Seca LMA , et al. (2019) Current trends on seaweeds: Looking at chemical composition, phytopharmacology, and cosmetic applications. Molecules 24(22): 4182. 10.3390/molecules24224182 PMC6891420 31752200

[bibr43-02601060251401628] Shannon E Abu-Ghannam N (2019) Seaweeds as nutraceuticals for health and nutrition. Phycologia 58: Article 5.

[bibr44-02601060251401628] Statistics Canada (2022) *The Canadian census: A rich portrait of the country's religious and ethnocultural diversity*. https://www150.statcan.gc.ca/n1/daily-quotidien/221026/dq221026b-eng.htm.

[bibr45-02601060251401628] Super S Wagemakers A (2021) Understanding empowerment for a healthy dietary intake during pregnancy. International Journal of Qualitative Studies on Health and Well-Being 16(1): 1857550. 10.1080/17482631.2020.1857550 PMC7738301 33317432

[bibr46-02601060251401628] Tremblay A Beaulieu L (2021) Extraction technologies for proteins and peptides. In: Recent Advancements in Micro and Macroalgal Processing: Food and Health Perspectives. Hoboken, NJ: Wiley-Blackwell, pp. 141–162.

[bibr47-02601060251401628] United Nations (2019) World Population Prospects 2019: Highlights. New York, NY: Department of Economic and Social Affairs, Population Division.

[bibr48-02601060251401628] Van Krimpen M Bikker P Van der Meer I , et al. (2013) *Cultivation, processing and nutritional aspects for pigs and poultry of European protein sources as alternatives for imported soybean products* (1570-8616).

[bibr49-02601060251401628] WHO (2016) *WHO recommendations on antenatal care for a positive pregnancy experience*. https://iris.who.int/bitstream/handle/10665/250796/9789241549912-eng.pdf?sequence=1.

[bibr50-02601060251401628] Xiao X Liu S Li L , et al. (2024) Seaweeds as a major source of dietary microplastics exposure in East Asia. Food Chemistry 450: 139317. 10.1016/j.foodchem.2024.139317 38636378

[bibr51-02601060251401628] Young M Paul N Birch D , et al. (2022) Factors influencing the consumption of seaweed amongst young adults. Foods (Basel, Switzerland) 11(19): 3052. 10.3390/foods11193052 PMC9563983 36230128

[bibr52-02601060251401628] Zimmermann M Delange F (2004) Iodine supplementation of pregnant women in Europe: A review and recommendations. European Journal of Clinical Nutrition 58(7): 979–984. 10.1038/sj.ejcn.1601933 15220938

