# Supplementary material for: Exploring the acceptability of seaweed consumption among pregnant individuals
Source: Nutr Health. 2025 Dec 8;32(6):1921–9. doi: 10.1177/02601060251401628 (PMC13744583; doi:10.1177/02601060251401628)
Supplement: Supplementary file 1 [file sj-docx-1-nah-10.1177_02601060251401628.docx]

**SUPPLEMENTARY MATERIALS**

**Figure S1**. Flow chart of participation inclusion

**ORIGINAL FRENCH VERSION**

**Questionnaire sur l’acceptabilité de la consommation d’algues chez les personnes enceintes**

*Lisez attentivement les questions suivantes et répondez au meilleur de vos connaissances.*

**Partie 1 – Caractéristiques personnelles**

1. **Quel âge avez-vous ? _____________**
2. **À combien de semaines de grossesse êtes-vous rendu(e) ? ___________________**
3. **Quel était le revenu total de votre ménage, avant impôt, l’année dernière?**

- 0 – 29 999 $
- 30 000 $ – 49 999 $
- 50 000 $ – 69 999 $
- 70 000$ – 99 999 $
- 100 000 $ – 149 999 $
- 150 000 $ ou plus
- Préfère ne pas répondre

4. **Quel est le niveau d’étude le plus élevé que vous avez complété ?**
   - Aucun diplôme
   - Diplôme d’études secondaires
   - Diplôme ou certificat d’une école de métier
   - Diplôme collégial ou du cégep
   - Certificat universitaire inférieur au baccalauréat
   - Baccalauréat
   - Diplôme ou certificat universitaire supérieur au baccalauréat
5. **Laquelle des catégories suivantes correspond le mieux à vos habitudes alimentaires ?**
   - Omnivore (consommation de viande, poisson, produits laitiers, œufs et végétaux/céréales/grains)
   - Végétarien (consommation de produits laitiers, œufs et végétaux /céréales/grains)
   - Végétalien (consommation de végétaux/céréales/grains).
6. **Est-ce que les algues (sushis, salade de wakamé, etc.) font partie de vos traditions alimentaires ou de votre culture ?**

- Oui
- Non

**Partie 2 – Attitude générale face à l’alimentation pendant votre grossesse**

**7. Nous aimerions connaître votre attitude envers votre santé et les aliments pendant votre grossesse. Pour chaque affirmation suivante, veuillez indiquer votre degré d’accord sur une échelle allant de « fortement en désaccord » à « fortement en accord ».**

|  | Fortement  en désaccord | Fortement  en accord | |
| --- | --- | --- | --- |
| a) Pendant ma grossesse, je suis très impliqué(e) au niveau de ma santé. | 1 2 3 4 5 | |  |
| b) Pendant ma grossesse, je suis très conscient(e) de ma santé. | 1 2 3 4 5 | |  |
| c) Pendant ma grossesse, l’aspect santé des aliments influence grandement mes choix alimentaires. | 1 2 3 4 5 | |  |
| d) Pendant ma grossesse, je suis très attentif(ve) aux valeurs nutritives des aliments que je consomme. | 1 2 3 4 5 | |  |

**8. Nous aimerions également connaître votre attitude face à de nouveaux aliments lors de votre grossesse. Pour chaque affirmation suivante, veuillez indiquer votre degré d’accord.**

|  | Fortement  en désaccord | | Fortement  en accord | |
| --- | --- | --- | --- | --- |
| a) Avant la grossesse, je goûtais souvent à de nouveaux aliments. | | 1 2 3 4 5 | |  |
| b) Pendant ma grossesse, j’accepte de goûter à de nouveaux aliments. | | 1 2 3 4 5 | |  |
| c) Pendant ma grossesse, j'ai peur de manger des aliments que je n'ai jamais mangés auparavant. | | 1 2 3 4 5 | |  |

**9. Répondez à l’affirmation suivante sur une échelle de 1 à 5 : Pendant ma grossesse, je suis conscient(e) de l’impact de mes choix alimentaires sur l’environnement.**

Fortement en désaccord	1	2	3	4	5	Fortement en accord

**Partie 3 – Consommation et perceptions des algues**

Les algues sont des végétaux aquatiques dont la plupart des espèces sont comestibles, telles que les algues brunes, rouges et vertes. Les algues font partie des traditions alimentaires de plusieurs pays d’Asie et la popularité de cet aliment augmente désormais en Europe et en Amérique du Nord. Les algues constituent d’ailleurs une ressource naturelle importante au Québec.

**10. En vous basant sur ce que vous connaissez des algues, quelle est votre attitude envers cette catégorie d'aliments ?**

Négative	Neutre	Positive

**11. Diriez-vous que manger des algues a des effets négatifs ou positifs pour votre santé ? Veuillez sélectionner une réponse ci-dessous.**

Négatifs	Neutres	Positifs

**12. Diriez-vous que manger des algues a des effets négatifs ou positifs pour la santé de votre bébé ? Veuillez sélectionner une réponse ci-dessous.**

Négatifs	Neutres	Positifs

**13. Croyez-vous que manger des algues est associé à des risques pour votre santé ?**

Oui	Peut-être	Non

**14. Croyez-vous que manger des algues est associé à des risques pour la santé de votre bébé ?**

Oui	Peut-être	Non

**15. Avez-vous déjà mangé des algues (peu importe la forme) pendant votre grossesse?**

Exemples : algues fraîches ou surgelées, produits à base d’algue (pesto, mélange à tartare), mets préparés qui contiennent des algues (sushis, salade, sandwich, pizza, soupe et grignotines).

- Oui (veuillez répondre aux questions 16 à 20)
- Non (veuillez répondre aux questions 21 et 22)

**16. Si oui, veuillez spécifier la ou les différentes formes : _______________________**

**17. Si oui, à quelle fréquence avez-vous mangé des algues pendant votre grossesse?**

- Tous les jours
- Quelques fois par semaine
- Une fois par semaine
- 2 à 3 fois par mois
- 1 fois par mois
- Quelques fois pendant la grossesse
- Une fois pendant la grossesse

**18. Si oui, quelle quantité d’algues avez-vous mangée chaque fois ?**

- 1 à 2 c. thé (5-10 ml)
- 1 à 2 c. à table (15-30 ml)
- 1/4 tasse (60 ml)
- 1/2 tasse (125 ml)
- 3/4 tasse (190 ml)
- 1 tasse (250 ml)
- Autres quantités (p.ex., unités) :

**19. Si oui, pour quelles raisons mangez-vous des algues ?**

- Pour les bénéfices liés à la santé
- Par tradition
- Pour le goût
- Pour faire différent, pour varier
- Pour d’autres raisons, nommez : ___________________

**20. Pensez-vous manger à nouveau des algues au cours de votre grossesse ?**

Oui	Peut-être	Non

**21. Si non, pour quelles raisons ne mangez-vous pas d’algues ?**

- À cause du goût
- Parce que je n’ai pas l’intérêt et/ou les connaissances nécessaires pour l’incorporer en cuisine
- En raison de la faible disponibilité des produits dans les commerces
- À cause du prix
- Pour d’autres raisons : nommez : __________________

**22. Si vous n’avez jamais consommé d’algues, seriez-vous prêt(e) à essayer un tel produit pendant votre grossesse ?**

Oui	Peut-être	Non

**Et pour quelles raisons ? Nommez : _______________________________________**

**23. Est-ce que vous voulez exprimer d'autres préoccupations ou opinions par rapport à la consommation d'algues pendant la grossesse?**

________________________________________________________________________

________________________________________________________________________

________________________________________________________________________

*Nous vous remercions de votre participation à ce projet de recherche.*

**ENGLISH TRANSLATION**

**Questionnaire on the acceptability of seaweed consumption among pregnant women**

*Read the following questions carefully and answer to the best of your knowledge.*

**Part 1 – Personal Characteristics**

1. **How old are you? _____________**
2. **How many weeks of pregnancy have you had? ___________________**
3. **What was your household’s total income, before tax, last year?**
   - 0 – 29 999 $

- 30 000 $ – 49 999 $
- 50 000 $ – 69 999 $
- 70 000$ – 99 999 $
- 100 000 $ – 149 999 $
- 150 000 $ or more
- Prefer not to answer

4. **What is the highest level of study you have completed?**

- No degree
- High school diploma
- Diploma or certificate from a trade school
- College or CEGEP diploma
- University certificate below baccalaureate
- Baccalaureate
- Diploma or certificate of higher education at the baccalaureate

5. **Which category best suits your eating habits?**
   - Omnivore (meat, fish, dairy, eggs and vegetables/grains/grain consumption)
   - Vegetarian (consumption of dairy products, eggs and vegetables/cereals/grains)
   - Vegan (consumption of vegetables/cereals/grains)
6. **Is seaweed (sushi, wakame salad, etc.) part of your food traditions or culture?**

- Yes
- No

**Part 2 – General attitude towards food during pregnancy.**

**7. We would like to understand your attitude toward your health and food during your pregnancy. For each of the following statements, please indicate your level of agreement on a scale ranging from 'strongly disagree' to 'strongly agree'.**

|  | Strongly disagree | Strongly agree | |
| --- | --- | --- | --- |
| a) During my pregnancy, I am very engaged in my health. | 1 2 3 4 5 | |  |
| b) During my pregnancy, I am very conscious of my health. | 1 2 3 4 5 | |  |
| c) During my pregnancy, the health aspect of food greatly influences my food choices. | 1 2 3 4 5 | |  |
| d) During my pregnancy, I pay close attention to the nutritional values of the food I consume. | 1 2 3 4 5 | |  |

**8. We would also like to understand your attitude toward new foods during your pregnancy. For each of the following statements, please indicate your level of agreement.**

|  | Strongly disagree | | Strongly agree | |
| --- | --- | --- | --- | --- |
| a) Before pregnancy, I often tried new foods. | | 1 2 3 4 5 | |  |
| b) During my pregnancy, I am willing to try new foods. | | 1 2 3 4 5 | |  |
| c) During my pregnancy, I am afraid of eating foods that I have never tried before. | | 1 2 3 4 5 | |  |

**9. Please respond to the following statement on a scale of 1 to 5: During my pregnancy, I am aware of the impact of my food choices on the environment.**

Strongly disagree	1	2	3	4	5	Strongly agree

**Part 3 – Consumption and perceptions of seaweed**

Seaweeds are aquatic plants, most species of which are edible, including brown, red, and green algae. Seaweeds are part of the food traditions in several Asian countries, and the popularity of this food is now increasing in Europe and North America. Seaweeds are also an important natural resource in Quebec.

**10. Based on what you know about seaweeds, what is your attitude toward this category of food?**

Negative	Neutral	Positive

**11. Would you say that eating seaweed has negative or positive effects on your health? Please select one of the responses below.**

Negative	Neutral	Positive

**12. Would you say that eating seaweed has negative or positive effects on your baby's health? Please select one of the responses below.**

Negative	Neutral	Positive

**13. Do you believe that eating seaweed is associated with risks to your health?**

Yes	Maybe	No

**14. Do you believe that eating seaweed is associated with risks to your baby's health?**

Yes	Maybe	No

**15. Have you ever eaten seaweed (in any form) during your pregnancy? Examples include fresh or frozen seaweed, seaweed-based products (pesto, tartare mix), prepared dishes containing seaweed (sushi, salad, sandwich, pizza, soup, and snacks).**

- Yes (please answer questions 16 to 20)
- No (please answer questions 21 and 22)

**16. If yes, please specify the different forms: ___________________________**

**17. If yes, how often have you eaten seaweed during your pregnancy?**

- Every day
- A few times a week
- Once a week
- 2 to 3 times a month
- Once a month
- A few times during the pregnancy
- Once during the pregnancy

**18. If yes, how much seaweed did you eat each time?**

- 1 to 2 teaspoons (5-10 ml)
- 1 to 2 tablespoons (15-30 ml)
- 1/4 cup (60 ml)
- 1/2 cup (125 ml)
- 3/4 cup (190 ml)
- 1 cup (250 ml)
- Other quantities (e.g., units):

**19. If yes, why do you eat seaweed?**

- For health benefits
- Out of tradition
- For the taste
- To be different, to vary
- For other reasons, please specify: ___________________

**20. Do you think you will eat seaweed again during your pregnancy?**

Yes	Maybe	No

**21. If no, why do you not eat seaweed?**

- Because of the taste
- Because I lack interest and/or knowledge to incorporate it into cooking
- Due to the low availability of products in stores
- Because of the price
- For other reasons, please specify: __________________

**22. If you have never consumed seaweed, would you be willing to try such a product during your pregnancy?**

Yes	Maybe	No

**And for what reasons? Please specify: _____________________________________**

**23. Would you like to express any other concerns or opinions regarding the consumption of seaweed during pregnancy?**

________________________________________________________________________

________________________________________________________________________

________________________________________________________________________

*We thank you for your participation in this study.*
